# Fitness change in relation to mutation number in spontaneous mutation accumulation lines of *Chlamydomonas reinhardtii*

**DOI:** 10.1111/evo.13360

**Published:** 2017-10-26

**Authors:** Susanne A. Kraemer, Katharina B. Böndel, Robert W. Ness, Peter D. Keightley, Nick Colegrave

**Affiliations:** ^1^ Institute of Evolutionary Biology, Ashworth Laboratories University of Edinburgh Edinburgh EH93FL United Kingdom; ^2^ Department of Biology, William G. Davis Building University of Toronto Mississuaga L5L1C6 Canada

**Keywords:** *Chlamydomonas*, mutation accumulation, mutational effects, spontaneous mutations

## Abstract

Although all genetic variation ultimately stems from mutations, their properties are difficult to study directly. Here, we used multiple mutation accumulation (MA) lines derived from five genetic backgrounds of the green algae *Chlamydomonas reinhardtii* that have been previously subjected to whole genome sequencing to investigate the relationship between the number of spontaneous mutations and change in fitness from a nonevolved ancestor. MA lines were on average less fit than their ancestors and we detected a significantly negative correlation between the change in fitness and the total number of accumulated mutations in the genome. Likewise, the number of mutations located within coding regions significantly and negatively impacted MA line fitness. We used the fitness data to parameterize a maximum likelihood model to estimate discrete categories of mutational effects, and found that models containing one to two mutational effect categories (one neutral and one deleterious category) fitted the data best. However, the best‐fitting mutational effects models were highly dependent on the genetic background of the ancestral strain.

Genetic variation originates from new mutations and selection acting on genetic variation ultimately leads to evolutionary change. The fitness properties of new, spontaneous mutations are therefore of interest in many areas of biology. For example, the rate of mutation per generation can determine the speed at which a population can adapt to changing environmental conditions (but see de Visser et al. 1999), and recombination is favoured because it increases the efficacy of selection against deleterious variants (Otto 2009; Hartfield et al. 2012). The majority of mutations affecting fitness seem to have a negative impact (Keightley and Lynch 2003) and the cumulative fitness impact of new mutations can be significant over evolutionary time scales (Eyre‐Walker and Keightley 1999; Lynch et al. 1999).

Although new mutations are of broad interest in evolutionary biology, their properties have been difficult to study directly. Any new mutation will be initially rare in a population, and in large populations, selection will be effective in removing deleterious mutations (Kimura and Ohta 1971). This implies that standing genetic variation for fitness is expected to result from the segregation of mutations with small deleterious effects. Consequently, population genetics approaches to determine the distribution of fitness effects of mutations are limited to that part of the distribution that segregates at appreciable frequencies within populations (Keightley and Eyre‐Walker 2010).

To obtain a more complete picture of the distribution of fitness effects of new mutations, mutation accumulation (MA) approaches have been widely applied. MA involves propagating multiple lines from a common ancestor under conditions where natural selection is minimized, with regular bottlenecking of the populations to one or very few individuals (see e.g., Halligan and Keightley 2009). After the period of MA, the fitness of MA lines can be compared to that of their ancestral genotypes to determine the cumulative impact of MA on fitness. With the exception of strongly deleterious mutations, the accumulation of mutations is expected to occur randomly in such experiments. MA experiments have been conducted in many species, ranging from prokaryotes to multicellular eukaryotes (for a review see Halligan and Keightley 2009). A common observation from these studies is that MA lines have higher variance and a lower mean fitness than their ancestors, the later indicating that the majority of mutations have a negative effect (e.g., Zeyl and De Visser 2001; Charlesworth et al. 2004; Morgan et al. 2014). However, the overall relationship between the number of mutations and the decline in fitness remains to be determined. Similarly, the relative importance of mutations in different parts of the genome (e.g., in coding vs noncoding DNA regions) on fitness is poorly understood.

Recently, decreasing costs of whole‐genome sequencing have allowed researchers to determine the number, type, and position of mutations throughout the genome of MA lines. In contrast to previous approaches, where mutational properties are inferred indirectly (Halligan and Keightley 2009), the comparison of MA lines with their ancestors allows the mutation rate to be estimated directly (Denver et al. 2012). In this study, we investigate previously generated MA lines of the single‐celled alga *Chlamydomonas reinhardtii* in which mutations have been characterized using whole genome sequencing. We directly examine the relationship between the numbers of mutation and fitness of MA lines. To increase the precision of the inferred relationships, we developed high throughput competitive assays, which allow us to measure fitness more accurately than has been previously possible in this system. We then combined this fitness information with sequence‐based information on the number, type, and position of mutations. We found that most mutations are either slightly deleterious or have no observable effect on competitive fitness (i.e., on growth in direct competition with a nonmutated line). The total number of mutations was significantly related to competitive fitness in comparison to the ancestor, an effect at least partially attributable by a significant negative impact of coding region‐located mutations on fitness. Lastly, to infer properties of the distribution of fitness effects of mutations, we modeled the relationship between fitness and the total number of mutations carried by each line.

## Methods

### STRAIN GENERATION AND MUTATION CALLING

The MA lines studied in this experiment were generated as described previously (Morgan et al. 2014). Briefly, *Chlamydomonas reinhardtii* strains (CC‐1373, CC‐1952, CC‐2342, CC‐2344, CC‐2931, and CC‐2937) were chosen from natural isolates collected between 1945 and 1993 (Morgan et al. 2014). These strains were ancestral to the MA lines (henceforth ancestor strains), and were grown up on standard Bold's agar medium. To initiate the MA experiment, 15 individual colonies of each ancestral strain were randomly chosen and transferred onto fresh Bold's agar plates (Bold 1942). Transfers between plates were then performed by randomly choosing one colony and spreading it on a new plate, thus bottlenecking each line to a single cell at each transfer, which is expected to minimize the effectiveness of natural selection. The interval between transfers was chosen to minimize selection against slow growing colonies (Morgan et al. 2014). This protocol was repeated until the MA lines had undergone approximately 1000 generations. At the end of the MA experiment, one randomly chosen colony per MA line was stored frozen.

As described in detail elsewhere (Ness et al. 2015a), we characterized the complement of mutations carried by each MA line by genome sequencing. Briefly, we sequenced DNA from each MA line using the Illumina GAII platform. The reads were aligned against the *C. reinhardtii* reference genome (version 5.3 (Merchant et al. 2007)) using BWA (Li and Durbin 2009) and genotypes were called with the UnifiedGenotyper of GATK (McKenna et al. 2010). The genotype information was then used to identify mutations of each MA line by comparison to its ancestor and to the other 14 MA lines derived from the same ancestor (Ness et al. 2015a). These SNPs and indels include 3490 nuclear mutations and 12 plastid mutations. No mutations were detected in the mitochondria (Ness et al. 2015b).

### MEASURING COMPETITIVE FITNESS

To detect mutations with small effects on fitness, precise measures are necessary. We employed high throughput flow cytometry to measure competitive fitness, that is fitness in comparison to a competitor genotype within the same well, based on direct cell counts (Gullberg et al. 2014). This method provides a number of advantages over growth rates based on optical density changes. First, competitor and focal genotype are grown within the same well, so micro gradients of environmental conditions during the experiment should impact them both similarly. Second, fitness measures based on competitive growth rate might provide a more meaningful fitness proxy than growth in isolation. Third, flow cytometry makes it possible to distinguish live single cells from dividing cells, debris, and even bacterial contaminants with high repeatability and fidelity.

We conducted fitness tests of all MA lines derived from five of the ancestral backgrounds (CC‐1952, CC‐2342, CC‐2344, CC‐2931, and CC‐2937) that were revivable after frozen storage. We excluded MA lines derived from the CC‐1373 ancestor, because we previously detected signatures of positive selection among these lines (Morgan et al. 2014). Since the fitness effect of a mutation may be environment‐specific, we assayed fitness in two environments, a benign environment comprising standard Bold's medium and in an environment known to be more stressful (Bold's medium supplemented with 2.5 g/L NaCl (Bell 1992)). We excluded two lines apparently containing hyper‐mutator mutations (containing >250 mutations: CC‐2344_L1 and CC‐2931_L5) and one apparent hypo‐mutator (containing only two mutations, CC‐1952_L4). The remaining 60 lines (carrying on average 58 mutations per line) were competed against the *C. reinhardtii* CC‐1690 lab strain marked with the Venus fluorescent protein, hereafter referred to as “competitor” or “Venus” (kindly provided by S. Mayfield). Venus excites at 515 nm and emits at 528 nm and can be clearly distinguished from *C. reinhardtii* autofluorescence (Rasala *et al*. 2013).

We inoculated samples of each MA line growing on solid Bold's medium into two 96‐well plates filled with 200 μL of liquid Bold's medium. At the same time, we inoculated six to seven pseudoreplicates for each of the six ancestor strains into wells of the same plates such that each plate contained at least one pseudoreplicate of each ancestor. The competitor was inoculated twice into 5 mL Bold's medium in a six‐well plate at the same time to obtain a sufficient amount of competitor culture for all competition assays. All cultures were grown shaken for four days at 25°C at 80% relative humidity.

To precondition the cultures to assay conditions, we diluted 20 μL samples from each MA line and ancestor strain culture in 180 μL of Bold's medium and into Bold's medium supplemented with 2.5 g/L of NaCl. The two Venus cultures were combined, then split into three pseudoreplicates for each environmental condition by adding 500 μL to 4.5 mL of either Bold's or Bold's supplemented with 2.5 g/L NaCl. All cultures were incubated for three more days under the same conditions as described previously.

On the start day of the growth assay, all Venus pseudoreplicates within each environmental condition were mixed to create a homogenous competitor culture acclimated to each test condition. For the competition assay, we created mixtures of each test culture and the Venus culture by combining 30 μL of each. To initiate the assay, 10 μL of each mixture or each pure MA test culture were added to 190 μL of Bold's or Bold's with 2.5 g/L NaCl. Within each assay plate, we additionally included two wells inoculated with 10 μL of Venus as a pure culture control to ensure that fluorescence emittance was stable over time. Each assay plate was duplicated and one randomly chosen plate per pair was used for the initial destructive cell counts while the other was incubated shaken at 25°C and 80% relative humidity for 72 hours. All cultures were then diluted 1:10 into fresh media to avoid entry into stationary phase and incubated for 24 more hours under identical conditions before being counted again.

### FLOW CYTOMETRY

Samples were analyzed using a FACSCanto II flow cytometer (Beckton Dickinson (BD) Immunocytometry Systems, UK) equipped with a 488‐nm argon laser and standard filter set‐up running FACSDiva 6 software. An electronic acquisition gate was applied to the Forward/Side scatter log‐plot around the chlorophyll positive population and 100,000 events were acquired in this gate. Chlorophyll was detected based on FL‐3 (670–735 nm) fluorescence emission and Venus was quantified based on FL‐1 (530 ± 30 nm) fluorescence emission. All particle counts were acquired using a BD High Throughput (HTS) system at 1 μL/s for 30 seconds with a threshold rate <10,000 events/second from 96‐well plates. Analysis was performed using custom R scripts (an example script is deposited in Dryad, https://doi.org/10.5061/dryad.4sg14).

### DATA PROCESSING

The raw particle counts obtained from the flow cytometer were filtered according to the following parameters. To exclude cell clumps and fragments, we removed all particles with forward scatter area (FSC‐A—indicating cell size) values smaller than 50,000 and larger than 250,000. Cells in the process of division were excluded by removing particles with forward scatter width (FSC‐W—indicating the width of the forward scatter signal) values smaller than 50,000 and larger than 100,000. Debris was excluded by removing side scatter (SSC‐A—indicative of cell granularity or complexity) values above 250,000. Lastly, particles not containing chlorophyll were removed by excluding all particles corresponding to a PerCP‐Cy5‐5‐A (a fluorochrom with similar absorption and emission characteristics to chlorophyll) excitation of below 1000. We recorded the excitation values corresponding to the PerCP‐Cy5‐5‐A and FITC‐A fluorochroms for all cells, after experimentally determining that these axes are the most efficient at separating Venus‐fluorescent from nonfluorescent cells in pilot studies.

We log transformed all PerCP‐Cy5‐5‐A and FITC‐A excitation values before further data processing. To estimate the number of Venus‐fluorescent and nonfluorescent cells within each competition well, we randomly sampled 500 data points from each dataset from pure cultures (the pure Venus wells on the plate, as well as the pure MA line culture) to be used as a training dataset. We trained a quadratic discriminant analysis model on this dataset to assign data points to two groups: MA line and fluorescent competitor. The model uses data point identity (MA line or competitor) as the response variable and the log transformed PerCP‐Cy5‐5‐A and FITC‐A excitation values as the predictor variables. The resulting model was then utilized to assign the cells within the corresponding competitive assay wells to either the MA line or the competitor. We validated each model by utilizing it to reassign the training dataset (where the origin of each data point was known) to the two parent strains and subsequently evaluating the number of correct identifications. On average, quadratic discriminant analysis models assigned 99.3% of data points to the correct group. We discarded all assays in which the discriminant function analysis failed to predict the identity of more than 5% of the training data set correctly (0.05% of all models), as this might indicate irregular fluorescence within the well. Furthermore, we discarded assays where the model failed to converge (2.6% of all models), as well as those were growth failed in either the competition well or one of the two corresponding pure culture wells (4.8%). See Fig. S1 for examples of both a training data set (panels A and B) and a corresponding mixed culture with the groups assigned (panel C).

The correlations between fitness measures of each MA line between flow cytometric replicates were consistently higher than the correlations between line fitness measures based on replicate OD measurements published previously (Morgan et al. 2014, replicate 1 – 2: *r^2^* = 0.515 ± 0.168 95% confidence interval, replicate 1 – 3: *r^2^* = 0.577 ± 0.155 95% confidence intervals, replicate 2 – 3: *r^2^* = 0.573 ± 0.156 95% confidence intervals, compared to a correlation of 0.4075 ± 0.201 95% confidence intervals from Morgan et al. 2014).

### CALCULATION OF COMPETITIVE FITNESS

We used the cell counts obtained from flow cytometry to calculate “competitive fitness,” a measure of the fitness of each line in direct competition with the Venus competitor within the same well. Firstly, we calculated the growth rates per hour of focal strains (*r_MA_*) or fluorescence‐marked competitors (*r_V_*) as:

(1) r= ln 10N96/N0/96,

where *N_96_* is the number of cells counted after 96 hours and *N_0_* is the number of cells counted at the beginning of the assay, that is at time point zero. The multiplication factor of 10 in the equation accounts for the 1:10 dilution done at 72 hours to keep cultures in exponential phase during the assay. The competitive fitness of each MA line was subsequently calculated as the difference between the Venus and MA line growth rates:

(2) $$w_{MA}=r_{MA}-r_{V}.$$

Likewise, the competitive fitness of an ancestor (*w_ANC_*) was calculated as:

(3) $$w_{ANC}=r_{ANC}-r_{V}.$$

We calculated the selection coefficient (*s*), as:

(4) $$s=w_{ANC}-w_{MA}.$$

Additionally, we calculated relative fitness (1 – *s*) to describe the competitive fitness of an MA line relative to the competitive fitness of its ancestor. Results based on relative fitness measures were similar to those based on competitive fitness and can be found in the supplemental statistics file. To aid comparisons across studies, we additionally calculated selective effects scaled by ancestral generation time (*s*_τ_) (Supplemental methods, Chevin 2011; Kraemer et al. 2016).

### STATISTICAL ANALYSIS

All statistical analyses were conducted using R (R Development Core Team 2009). We utilized linear‐mixed models assuming a normal error distribution, as implemented in the packages nlme and lme4 (Bates et al. 2015; Pinheiro et al. 2016) to investigate the impact of the number and classes of mutations of each MA line on its competitive and relative fitness. The genetic background of each MA line was included as a random effect on the intercept. Ancestors of MA lines were included as lines with zero mutations in all models. Because we were hypothesis‐testing the impact of different mutational properties on fitness, we did not perform a sequential model fitting. All model details can be found in Tables 2 and 3 and the supplemental statistics.

### INFERENCE OF THE DISTRIBUTION OF EFFECTS OF MUTATIONS ON COMPETITIVE FITNESS

To investigate if there are models for the distribution of fitness effects (DFE) of new mutations that can explain the observed patterns of changes in fitness among lines, we developed a maximum likelihood approach to estimate DFE parameters based on competitive fitness estimates and the numbers of mutations carried by each line, the latter inferred by genome sequencing (Ness et al. 2015a).

Let *X_i_* be the estimated competitive fitness for MA line or control replicate *i* of a given ancestral strain (corresponding to the fitness measure *w_MA_* or *w_Anc_*), and *n_i_* be the number of mutations carried by that MA line, with *n_i_* = 0 for an ancestor genotype. Following Kousathanas and Keightley (2013), rather than fitting a continuous distribution for the DFE, we fitted models incorporating *c* categories (*c* ≥ 1) of discrete mutational effects **s** = [*s_1_, s_2_ … s_c_*]. This typically gives a superior fit to the data than a parametric distribution, such as the gamma distribution. We assumed an additive model, increasing the number of categories until there was no improvement in model fit (likelihood ratio tests, *P* > 0.05). We assumed that the first category of mutational effects had no effect on fitness, that is *s_1_* = 0 (“neutral”), and we estimated the fitness effects associated with the remaining *c* – 1 categories. The proportions of mutational effects in each category were specified by a vector **p** = [*p_1_, p_2_…p_c_*], where *p_1_* is the proportion of the mutational effect that has no effect on fitness, and Σ*p* = 1. There are therefore *c* – 1 proportions to be estimated in the model. We assumed that the mutations are independently distributed among the categories, that is multinomially distributed *f*(*c*, **p**, **n**), where **n** = [*n_1_, n_2_…n_c_*] is a vector of the numbers of mutations in the different categories carried by an MA line, with Σ*n* = *n_i_*. Taking a model with *c* = 2 categories of mutational effects as an example, the likelihood for observation *X_i_* is:

(5) $$L_{i}=\sum_{j=0}^{n_{i}}\sum_{k=0}^{n_{i}-j}f\left(2,p,n\right)\Phi \left(X_{i}-ks_{2},\mu ,V_{E}\right),$$

$$p=\left[p_{1},1-p_{1}\right],\;n=\left[j,k\right]$$

where Φ(*Y*, *μ*, *V_E_*) is the density of the normal distribution probability density function at point *Y*, *μ* = the mean for the control or unmutated lines and *V_E_* = the environmental variance (containing the error variance). Thus, out of *n* mutations detected in a given MA line, *k* will belong to the second mutational effects category and will reduce fitness by a factor of *s_2_* each. There are similar equations for *c* = 1 (a null model of only neutral mutations) and *c* = 3 (Supplementary information). For each model, we estimated *μ*, *V_E_*, the proportion of the mutational effect categories, as well as their respective selective effects.

We also tested models with an additional parameter, Δ*V_E_*, which allows for the residual variance to change linearly with the number of mutations. The addition of this parameter did not improve the model fit for one or two effects category models (based on likelihood ratio tests). We present the results of these models in Table S1.

The overall likelihood across *m* independently generated MA lines of a given ancestral genotype was:

(6) $$logL=\sum_{x=1}^{x=m}logL_{X}.$$

### MAXIMIZATION OF LOG LIKELIHOOD

Likelihood was maximized using the simplex algorithm (Nelder and Mead 1965). The model potentially has a large number of parameters and there is the possibility of local likelihood maxima, which the simplex algorithm might find rather than the global maximum. To find the global maximum log likelihood for each *c*, for each strain we estimated the parameters of the model between 20 and 250 times using varying starting values until a plateau log likelihood value was reached. Starting values were randomly chosen from the following ranges: *μ*: mean competitive fitness ± 0.015, *V_E_*: replicate variance ± 0.00005, Δ*V_E_*: –0.001 – 0.001, *s_2_… s_c_*: –0.5 – 0.5, and *p_2_…p_c_*: 0 – 1 with Σ*p_2_…p_c_* < 1 so that *p_1_* = 1 – Σ*p_2_…p_c_*.

## Results

### COMPARING COMPETITIVE FITNESS WITH FITNESS MEASURES BASED ON GROWTH RATES IN ISOLATION

**Figure 1:**
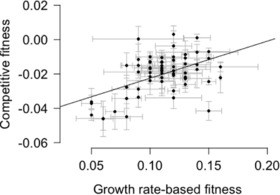
Relationship between competitive fitness measures and growth rate‐based fitness calculated [data from Morgan et al. (2014)]. Error bars indicate standard errors of the mean.

Competitive fitness (*w_MA_*) and fitness based on growth rates in isolation (data previously published in Morgan et al. 2014) were highly and significantly correlated (Pearson's product‐moment correlation coefficient of competitive fitness with fitness calculated via growth rates: 0.502, *P* = 2.03 × 10^5^, Fig. 1). Similarly, relative fitness values calculated based on competitive fitness (1 – *s*) and on growth rates in isolation were significantly correlated (Pearson's product‐moment correlation coefficient: 0.604, *P* = 1.01 × 10^−7^, Fig. S2).

### SELECTIVE EFFECT OF MUTATIONS IN BENIGN CONDITIONS

To estimate the average selective effect of an individual mutation, we divided the total selective effect of all mutations (*s*) of each line by the total number of mutations it carried (including SNPs and indels). The average selective effect (*s*) per mutation and hour across all MA lines and genetic backgrounds in benign conditions was 0.000101 (± 0.0000541 standard error, Table 1).

**Table 1:** Average selective effect estimates (*s*) based on competitive fitness, average selective effect scaled by generation time (*s_τ_*) per mutation, selective effect per mutation based on growth rates and scaled selective effect based on growth rate (Morgan et al. 2014) ± standard errors, by genetic background and across all MA backgrounds

Ancestral genotypes	Number of MA lines	*s* per mutation (competitive fitness)	*s_τ_* per mutation (competitive fitness)	*s* per mutation (growth rate)	*s_τ_* per mutation (growth rate)
CC‐1952	13	0.0000732 ± 0.0000977	0.000775 ± 0.00143	0.00126 ±0.000358	0.00609 ±0.00177
CC‐2342	10	0.000393 ± 0.000224	0.00481 ±0.00281	0.000984 ±0.000535	0.00429 ±0.00248
CC‐2344	12	0.000165 ± 0.0000949	0.00193 ±0.00134	0.000406 ±0.000258	0.00258 ±0.00165
CC‐2931	11	0.000119 ± 0.0000235	0.00144 ±0.000305	0.000229 ±0.0000465	0.00101 ±0.000216
CC‐2937	14	–0.000151 ± 0.0000859	–0.00340 ±000176	–0.000487 ±0.000162	–0.00589 ±0.000137
All backgrounds	60	0.000101 ± 0.0000542	0.000827 ±0.00800	0.000447 ±0.000154	0.00115 ±0.000896

Selective effects per mutation varied significantly between MA lines derived from different genetic backgrounds (ANOVA of selective effects as a function of genetic background, *P* = 0.031). This result was largely driven by one genetic background, CC‐2937, in which the net effect of *s* was negative, indicating an increase in fitness. This contrast with the other genetic backgrounds, in which selective effects were all positive, causing a decrease in fitness (Table 1). Selective effects scaled by generation time (*s*_τ_) mimic these patterns (Table S1).

The selective effects per mutation calculated here are on average smaller in magnitude than those calculated based on growth in isolation (data previously published in Morgan et al. 2014, Table 1). However, this difference does not persist when we compare selective effects scaled by generation time between the two datasets (Table 1), indicating a difference in generation time between assays.

### MUTATIONAL EFFECTS ON FITNESS IN BENIGN CONDITIONS

**Figure 2:**
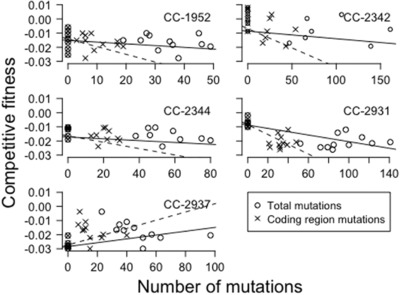
Competitive fitness plotted against the total number of mutations (open circles and solid lines) and the total number of coding region mutations (crosses and dashed lines) in the five genetic backgrounds.

Competitive fitness of the MA lines was significantly lower than that of their respective ancestors (linear‐mixed model, genetic background as random effect, *P* < 0.05, Table 2: model 1) and the total number of mutations carried showed a significantly negative relationship with competitive fitness (linear‐mixed model, genetic background as a random effect, *P* < 0.05, Table 2: model 2, Fig. 2: open circles and solid lines). In contrast, competitive fitness was neither significantly impacted by the number of SNPs and indels (all *P* > 0.05, Table 2: model 3), nor by the number of synonymous or nonsynonymous mutations (all *P* > 0.05, Table 2: model 4, all linear‐mixed models, genetic background as a random effect).

**Table 2:** Fixed effects, regression coefficients, and *P*‐values of models with competitive fitness as the response variable

Model number	Fixed effect	Regression coefficient	*P*‐value
1	MA line or ancestor	–0.00751	2.1 × 10^−5***^
2	Total number of mutations	–5.326 × 10^–5^	0.024^*^
3	Number of SNPs	–4.239 × 10^–5^	0.22
	Number of indels	–1.035 × 10^–4^	0.38
4	Nonsynonymous mutations	–2.471 × 10^–4^	0.13
	Synonymous mutations	–1.721 × 10^–4^	0.58
5	Exonic mutations	–3.819 × 10^–4^	0.00080^***^
	Intronic mutations	3.799 × 10^–4^	0.035^*^
	Intergenic mutations	3.426 × 10^–4^	0.40
6	Intergenic mutations	2.338 × 10^–4^	0.57
	Intronic mutations	3.898 × 10^–4^	0.029^*^
	CDS‐located mutations	–6.497 × 10^–4^	0.00014^***^
	UTR‐located mutations	3.784 × 10^–5^	0.87

Genetic background was included as a random effect in all models. More model details can be found in the supplemental information.

When partitioning the mutations into coding (exonic) and noncoding (intergenic and intronic) mutations, we detected a significantly negative impact of mutations located within exons on competitive fitness (*P* < 0.001, Table 2: model 5, Fig. S3: circles and solid lines). Surprisingly, intronic mutations had a slight, but significantly positive impact on competitive fitness. However, this was exclusively due to MA lines derived from a single genetic background, CC‐2937 (*P* < 0.05, Table 2: model 5, Fig. S3: crosses and dashed lines). We tested the effect of mutational location further by partitioning the exonic region into coding and untranslated regions (UTR). On average, MA lines carried 20 mutations within their coding regions (CC‐1952: 11 mutations, CC‐2342: 26, CC‐2344: 20, CC‐2931: 33, CC‐2937: 15). We found a significantly negative effect on competitive fitness of the number of mutations located within coding regions, but no significant effect in the case of UTR‐located mutations (linear‐mixed model, genetic background as random effect, *P* < 0.001 and *P* > 0.05, respectively, Fig. 2: crosses and dashed lines, Table 2: model 6). Additionally, we recovered the significantly positive impact of intron‐located mutations (*P* < 0.05, Table 2: model 6, Fig. S3: crosses and dashed lines).

### ARE MUTATIONAL EFFECTS INCREASED UNDER STRESSFUL CONDITIONS?

It has been suggested that stressful conditions might increase the mean effects of deleterious mutations or the amount of new mutational variation (Remold and Lenski 2001; Cooper et al. 2005; Baer et al. 2006; Martin and Lenormand 2006). To further investigate the effect of stress on mutational effects, we conducted competitive fitness assays in medium supplemented with 2.5 g/L NaCl, representing moderate stress (Kraemer et al. 2015). Moderately stressful conditions represent a more realistic scenario for environmental conditions that might be encountered by new mutants, in contrast to nearly lethal conditions. Overall, competitive fitness was slightly, but not significantly higher in moderately stressful than in benign conditions (linear‐mixed model, genetic background as random effect, *P* = 0.052, Table 3: model 1). This indicates that the common competitor genotype may be more strongly impacted by stressful conditions than the MA lines and their ancestors. While MA lines are less fit than their ancestors, we did not find their fitness to be impacted by the stress treatment (*P* < 0.05 and *P* > 0.05, respectively, Table 3: model 1).

**Table 3:** Fixed effects, regression coefficients, and *P*‐values of models investigating the effect of moderate stress with competitive fitness as the response variable

Model number	Fixed effect	Regression coefficient	*P*‐value
1	Treatment	4.620 × 10^−3^	0.052
	MA line or ancestor	–7.479 × 10^−3^	0.00028^***^
	Treatment × type	1.621 × 10^−5^	0.10
2	Treatment	5.337 × 10^−3^	0.0080^**^
	Total number of mutations	–6.614 × 10^−5^	0.013^*^
	Treatment × total number of mutations	–1.690 × 10^−5^	0.65
3	Treatment	5.306 × 10^−3^	0.0084^**^
	Number of SNPs	–5.663 × 10^−5^	0.14
	Number of indels	–1.091 × 10^−4^	0.41
	Treatment × SNPs	–6.350 × 10^−5^	0.23
	Treatment × indels	1.997 × 10^−4^	0.27
4	Treatment	4.962 × 10^−3^	0.012^*^
	Number of nonsynonymous mutations	–2.940 × 10^−4^	0.12
	Number of synonymous mutations	–2.008 × 10^−4^	0.58
	Treatment × nonsynonymous mutations	–2.110 × 10^−4^	0.42
	Treatment × synonymous mutations	3.470 × 10^−4^	0.50
5	Treatment	5.165 × 10^−3^	0.0097^**^
	Exonic mutations	–4.375 × 10^−4^	0.00061^***^
	Intronic mutations	4.215 × 10^−4^	0.039^*^
	Intergenic mutations	3.897 × 10^−4^	0.41
	Treatment × exonic mutations	1.171 × 10^−4^	0.50
	Treatment × intronic mutations	–4.528 × 10^−4^	0.12
	Treatment × intergenic mutations	7.719 × 10^−4^	0.25
6	Treatment	5.007 × 10^−3^	0.012^*^
	Intergenic mutations	3.029 × 10^−4^	0.52
	Intronic mutations	4.379 × 10^−4^	0.034^*^
	UTR‐located mutations	–5.899 × 10^−5^	0.81
	CDS‐located mutations	–6.880 × 10^−4^	0.00041^***^
	Treatment × intergenic mutations	7.810 × 10^−4^	0.25
	Treatment × intronic mutations	–4.535 × 10^−4^	0.12
	Treatment × UTR mutations	–5.344 × 10^−5^	0.88
	Treatment × CDS mutations	2.371 × 10^−4^	0.39

Genetic background was included as a random effect in all models. More model details can be found in the supplemental information.

The mean selective effect per mutation under stressful conditions was 0.0000178 (± 0.0000561 standard error). However, mean *s* per mutation did not differ significantly between benign and stressful conditions (paired Student's *t*‐tests for *s* per mutation per MA line in benign and stressful conditions, *P* = 0.10). Likewise, we did not detect any differences in the new mutational variation between benign and stressful conditions (Levene's test for *s* per mutation per MA line in benign and stressful conditions, *P* = 0.705).

The stress treatment did not impact the effect of mutations on the competitive fitness of MA lines (Table 3). While we recovered the negative effects of the total number of mutations, of exonic and CDS‐located mutations, as well as the significantly positive effect of intronic mutations (*P* < 0.05, Table 3: model 2, model 5, model 6), none of these effects were significantly impacted by moderate stress (*P* > 0.05, Table 3: model 2, model 5, model 6).

### INFERENCE OF DISTRIBUTIONS OF MUTATIONAL EFFECTS FOR FITNESS

We investigated the fit of models with different numbers of categories of mutational effects to our fitness data. The different genetic backgrounds showed significantly different fitness trajectories, so this analysis was performed independently for each genetic background. The best‐fitting models were determined via likelihood ratio tests (LRTs) (Table 4). In two cases (CC‐2342, CC‐2937), models with one category of mutational effects (*c* = 1, indicating no significant fitness impact of mutations (i.e., only neutral mutations)), fitted the data best. For MA lines derived from the other three genetic backgrounds, models with two effect categories (*c* = 2) fitted significantly better than models with just a single category, suggesting that these genetic backgrounds have at least one category of mutational effects impacting fitness under the assay conditions (Table 4, all *P* < 0.05). Adding an additional category of mutational effects (*c* = 3) did not improve model fit significantly for any of those datasets (Table 4, all *P* > 0.05). Incorporating the variance parameter Δ*V_E_*, which allows the residual variance to change linearly with mutation number, did not improve model fits, based on likelihood ratio tests (Table S1, Fig. S4). This result is consistent with a lack of detectable relationship between the variance and the mean fitness of a line (Fig. S5).

**Table 4:** Relative log likelihoods for models with different numbers of mutational effect categories for the five genetic backgrounds

Genetic background	*c*	*Relative logL*	LRT to models with less effect categories (*P*‐value)
CC‐1952	1	–3.1203	
**CC‐1952**	**2**	**–0.00255**	0.044^*^
CC‐1952	3	0	1.00
**CC‐2342**	**1**	**–4.203**	
CC‐2342	2	–1.422	0.062
CC‐2342	3	0	0.24
CC‐2344	1	–4.0533	
**CC‐2344**	**2**	**–0.131**	0.020^*^
CC‐2344	3	0	0.32
CC‐2931	1	–8.00563	
**CC‐2931**	**2**	**–0.443**	0.00052^***^
CC‐2931	3	0	0.064
**CC‐2937**	**1**	**–2.0453**	
CC‐2937	2	0	0.13
CC‐2937	3	0	1.00

The best‐fitting model for each background is indicated in bold. To obtain *P*‐values, we used a chi‐square distribution with degrees of freedom equal to the number of additional parameters added.

**Figure 3:**
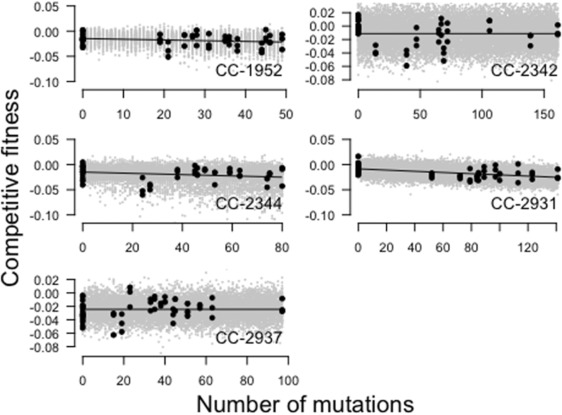
Competitive fitness plotted against the total number of mutations in the five genetic backgrounds. Black dots represent observed fitness values, gray dots represent predicted fitness values based on the frequencies of mutational effect categories derived from the best‐fitting model of mutational effect categories (Table 3). Black lines indicate a linear model fit of the observed data.

To visualize the fit of the models, we simulated data based on the best‐fitting models and plotted it along the observed fitness data (Fig. 3, observed data: black circles, simulated data: gray circles). Simulated fitness values based on the best‐fitting model of mutational effects corresponded well to the observed data.

Likewise, we investigated models with different mutational effect categories of exonic mutations only (Table S2). In this case, we detected a significant deleterious effect category for lines derived from the CC‐2342 background. Moreover, the proportion of deleterious mutations was higher for MA lines from the backgrounds CC‐1952, CC‐2344, and CC‐2931.

### MUTATIONAL EFFECT CATEGORIES

All mutational effect categories impacting fitness were deleterious. Somewhat surprisingly though, the mutational effects estimated were quite small (*s* < 0.1). Mutational effects were also highly dependent on the genetic background of the MA lines investigated. For example, we observed differences in the proportions of mutations with and without detectable fitness effects among the strains (Table 5). While our dataset does not include a wide enough range of genetic backgrounds to draw systematic conclusions about the relationship between the similarity of mutational effect class proportions and relatedness, it is noteworthy that the mutational effect categories detected vary widely even between two very closely related genetic backgrounds (CC‐2342 and CC‐2344, Flowers et al. 2015).

**Table 5:** Maximum likelihood parameter estimates (± 95% confidence intervals) for each strain for best‐fitting models of numbers of mutational effect categories

Strain	*c*	*p_1_*	*s_1_*	*p_2_* (*± 95% confidence intervals*)	*s_2_* (*±* 95% confidence intervals)
CC‐1952	2	0.992	0	0.00815 (0.00126, 0.637)	–0.0178 (–0.0313, –0.000194)
CC‐2342	1	1	0		
CC‐2344	2	0.996	0	0.00408 (0.00149, 0.00809)	–0.0310 (–0.0395, –0.0193)
CC‐2931	2	0.886	0	0.114 (0.0134, 1)	–0.000105 (–0.00744, –0.0000609)
CC‐2937	1	1	0		

*c*, number of categories of mutational effects, *p* – proportion, ***s*** – selection coefficient.

## Discussion

Directly determining the fitness effects of new mutations has been a long‐standing goal in evolutionary biology (Kondrashov 1988; Otto 2009). Lines derived in MA experiments offer the opportunity to study the fitness effects of all but the most deleterious of mutations, and thus to directly assess traits such as the mutation rate (Baer et al. 2006; Ness et al. 2015a) and the distribution of fitness effects of mutations (Halligan and Keightley 2009). Most mutational effects across the genome are expected to be either neutral or very mildly deleterious (Keightley and Lynch 2003). Moreover, such effects may be strongly influenced by the environment in which they are measured (Martin and Lenormand 2006). To study mutations of small effect, highly accurate fitness measures are necessary. Here, we utilized flow cytometry to obtain such fitness measures to determine the effects of new mutations in the green algae *C. reinhardtii*. This study thus connects a fine scale fitness analysis of MA lines within detailed sequence information about the number, type, and position of the causal mutations.

A long‐standing question in microbial experimental evolution has also been the extent by which fitness based on growth rates in isolation proxies the overall evolutionary fitness of a genotype (i.e., the probability that a newly arising mutant will outcompete its ancestor (Hall et al. 2014; Vale et al. 2015)). We estimated the correlation between competitive fitness measured in this study with a growth rate‐based fitness measure from a previous study of the same MA lines (Morgan et al. 2014). Overall, the two fitness measures, as well as the respective derived relative fitness measures, were highly significantly and positively correlated, indicating that competitive fitness can be, to some degree, compared across studies to previous fitness measures obtained via growth rates in isolation (e.g., Kassen and Bell 2000; Morgan et al. 2014; Lachapelle et al. 2015). However, we found that competitive fitness measures consistently have higher inter‐replicate correlations and smaller 95% confidence intervals than growth rate‐based fitness measures and are thus able to provide more precise estimates of small mutational effects.

In accordance with previous results (Zeyl and De Visser 2001; Charlesworth et al. 2004; Baer et al. 2006; Morgan et al. 2014), lines that have accumulated mutations under reduced selection suffered a reduction in fitness. Moreover, lines are generally less fit the more mutations they have accumulated. However, we failed to detect significant effects of the number of different molecular types of mutations (SNPs or indels) on fitness, although indels cause larger sequence disruptions than SNPs, and can cause frame shifts. The absence of a significant effect of indels may be due to their low overall number (∼10 per MA line) compared to the number of SNPs (48 per MA line).

A priori, we might expect the effects of mutations to be greatest in coding regions, compared to intergenic or intronic sites. Indeed, in an analysis restricted to this set of mutations, we did detect the expected negative relationship. Thus, much of the reduction in fitness seen in our MA lines appears to be due to mutations that fall in these regions. While not unexpected, this is, as far as we are aware, the first study to show this directly. Within a coding region, we found no evidence to suggest that this result was due specifically to the number of these mutations that were nonsynonymous. This might suggest that synonymous mutations may also have fitness consequences (e.g., Bailey et al. 2014), but more likely was simply due to a lack of statistical power. Intriguingly and unexpectedly, we also detected a significant positive effect of intronic mutations on fitness. However, this effect seems to be largely caused by a single genetic background (CC‐2937), which actually shows a slight fitness increase during MA, indicating that even though care was taken to minimize selection, lines derived from very slow growing ancestors might have accumulated beneficial mutations.

To further investigate mutational effects within our dataset, we used maximum likelihood to estimate the number of discrete mutational effect categories that best explain our data, and found that most genetic backgrounds were characterized by either one or two mutational effect categories. In two of the five genetic backgrounds, the best‐fitting model only allowed for neutral mutations. In the other three backgrounds a second, slightly deleterious effect category was fitted. Overall, the best‐fitting model represented a good fit to the actual fitness data (Fig. 3). As expected, the proportion of mutations in a deleterious effect class is increased if we focus the analysis on mutations more likely to impact fitness, such as exonic mutations.

The differences in mutational effects among the different genetic backgrounds opens up the possibility for the existence of genotype‐specific mutational trajectories (i.e., different genotypes may have different propensities to incur different categories of mutational effects potentially resulting in different evolutionary trajectories). However, while genotype‐specific trajectories have been outlined for the case in which different populations adapt to a fitness peak via beneficial mutations (e.g., different “starting points” of genotypes in Fisher's geometric model (Fisher 1930; Orr 2006)), such hypotheses are difficult to apply to “unselected” mutations derived from a MA experiment. It is notable that the closely related strains CC‐2342 and CC‐2344 were characterized by very dissimilar estimated frequencies of mutational effect categories and also varied in the total number of mutations found. However, a more thorough investigation of mutational effect categories across a range of relatedness is necessary to determine if the genetic architecture can influence the frequencies of mutational effect categories systematically.

It has been proposed that environmental stress might impact the selective effects of mutations and, for example, lead to a release of cryptic genetic variation that cannot be observed under benign conditions (Latta *et al*. 2015). Here, we focused our test on moderately stressful conditions (Kraemer *et al*. 2015), since these conditions represent a more realistic environmental stress than nearly lethal stress. The common competitor was on average more impacted by moderate stress than the MA lines are their ancestors. This reduced stress‐tolerance could be due to a longer cultivation period in the lab (CC‐1690 was isolated in 1955) or could be a cryptic cost of the genetic manipulation and fluorescent marking of the strain. We did not observe any significant impact of stress on the expression of mutational effects. Thus, in our study moderate stress did not lead to an exacerbation of mutational effects, nor did we detect differences in the variances of such effects across different environmental conditions (e.g., Martin and Lenormand 2006). Our findings contrast with other studies on the fitness effects of accumulated mutations in a range of model organisms such as *E. coli* (Cooper and Lenski 2000; Remold and Lenski 2001), yeast (Szafraniec *et al*. 2001; Jasnos *et al*. 2008), and *Drosophila* (Kondrashov and Houle 1994; Fry and Heinsohn 2002; Wang et al. 2009; Young *et al*. 2009). However, stress‐dependent fitness effects are far from general and their absence has been reported previously in the same systems (e.g. (Korona 1999; Kishony and Leibler 2003; Jasnos *et al*. 2008), and in a previous study on the same *C. reinhardtii* MA lines utilizing growth rates as a measure of fitness (Kraemer *et al*. 2015). Importantly, this result indicates that the mutational effects measured here can be extrapolated across different environments.

In summary, this study design allowed us to make direct connections between DNA sequence and fitness data to determine the impact of the number of new mutations on fitness. While MA lines were less fit on average than their ancestors, this fitness decline was largely unrelated to the type of mutation (SNPs or indels) carried by each individual MA line. In contrast, the number of mutations located within exonic and coding regions significantly and negatively impacted MA line fitness. Thus, most new mutations did not have observable fitness effects (at least under the environmental conditions utilized here) and overall decline in fitness was due to few mutations of detectable deleterious effects, many located within coding regions.

Associate Editor: T. Bataillon

Handling Editor: P. Tiffin

## Supporting information


**Figure S1**. Example of flow cytometry data plots and clustering of groups within mixed cultures. Each data point is plotted based on its PerCP‐Cy5‐5‐A and FITC‐A fluorescence. Upper panel: MA training dataset: 500 data points sampled randomly from all data points of the pure MA culture. Middle panel: Venus training data set: 500 data points randomly sampled from a pure Venus culture located on the same plate. Lower panel: Example of a mixed culture with group assignments based on the training data sets. Circles represent MA line cells, triangles Venus competitor cells within the same well.

**Figure S2**. Correlation between relative fitness values calculated from either competitive fitness or growth rate‐based fitness (based on changes in optical density, data obtained from Morgan *et al*., 2014). Error bars indicate standard errors of the mean.

**Figure S3**. Competitive fitness plotted against the total number of exonic mutations (open circles and solid lines) and the total number of intronic mutations (crosses and dashed lines) in the five genetic backgrounds.

**Figure S4**. Competitive fitness plotted against the total number of mutations in the five genetic backgrounds. Black dots represent observed fitness values, grey dots represent predicted fitness values based on the frequencies of mutational effect categories derived from the best‐fitting model of mutational effect categories incorporating the parameter Δ*V_E_* (Supplemental Table 1). Black lines indicate a linear model fit of the observed data.

**Figure S5**. Mean and variance of unscaled competitive fitness of each MA line. We did not detect a relationship between the mean and the variance of unscaled competitive fitness.

**Table S1**. Maximum likelihood parameter estimates for each strain for models of with one or two mutational effect categories, allowing for varying residual variance.

**Table S2**. Maximum likelihood parameter estimates for each strain for models of with one or two mutational effect categories, taking only into account the number of exonic mutations per MA line.

Supplemental material

supplemental_statistics
