# Supplementary figures and images for: Fitness change in relation to mutation number in spontaneous mutation accumulation lines of Chlamydomonas reinhardtii
Source: Evolution. 2017 Oct 26;71(12):2918–29. doi: 10.1111/evo.13360 (PMC5765464; doi:10.1111/evo.13360)

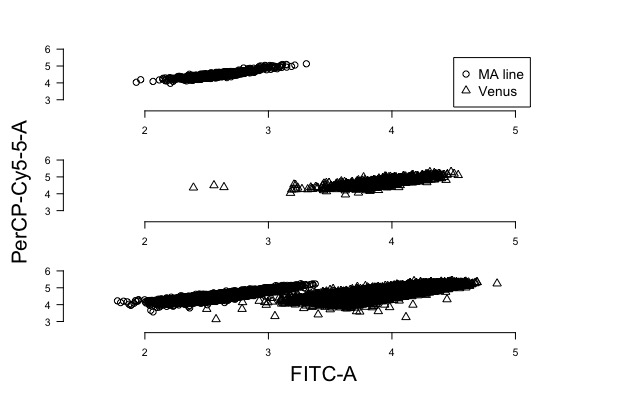

Supplement: Supplementary file 1 — Figure S1. Example of flow cytometry data plots and clustering of groups within mixed cultures. Each data point is plotted based on its PerCP‐Cy5‐5‐A and FITC‐A fluorescence. Upper panel: MA training dataset: 500 data points sampled randomly from all data points of the pure MA culture. Middle panel: Venus training data set: 500 data points randomly sampled from a pure Venus culture located on the same plate. Lower panel: Example of a mixed culture with group assignments based on the training data sets. Circles represent MA line cells, triangles Venus competitor cells within the same well. [file EVO-71-2918-s001.jpeg]

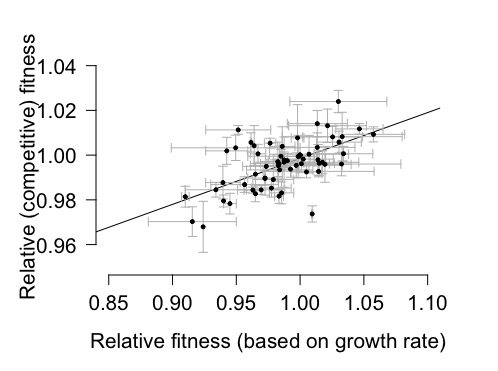

Supplement: Supplementary file 2 — Figure S2. Correlation between relative fitness values calculated from either competitive fitness or growth rate‐based fitness (based on changes in optical density, data obtained from Morgan et al., 2014). Error bars indicate standard errors of the mean. [file EVO-71-2918-s002.jpeg]

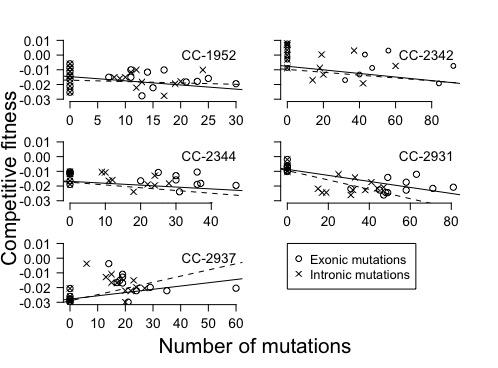

Supplement: Supplementary file 3 — Figure S3. Competitive fitness plotted against the total number of exonic mutations (open circles and solid lines) and the total number of intronic mutations (crosses and dashed lines) in the five genetic backgrounds. [file EVO-71-2918-s003.jpeg]

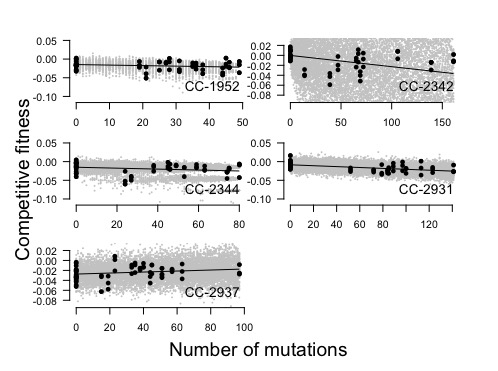

Supplement: Supplementary file 4 — Figure S4. Competitive fitness plotted against the total number of mutations in the five genetic backgrounds. Black dots represent observed fitness values, grey dots represent predicted fitness values based on the frequencies of mutational effect categories derived from the best‐fitting model of mutational effect categories incorporating the parameter ΔVE (Supplemental Table 1). Black lines indicate a linear model fit of the observed data. [file EVO-71-2918-s004.jpeg]

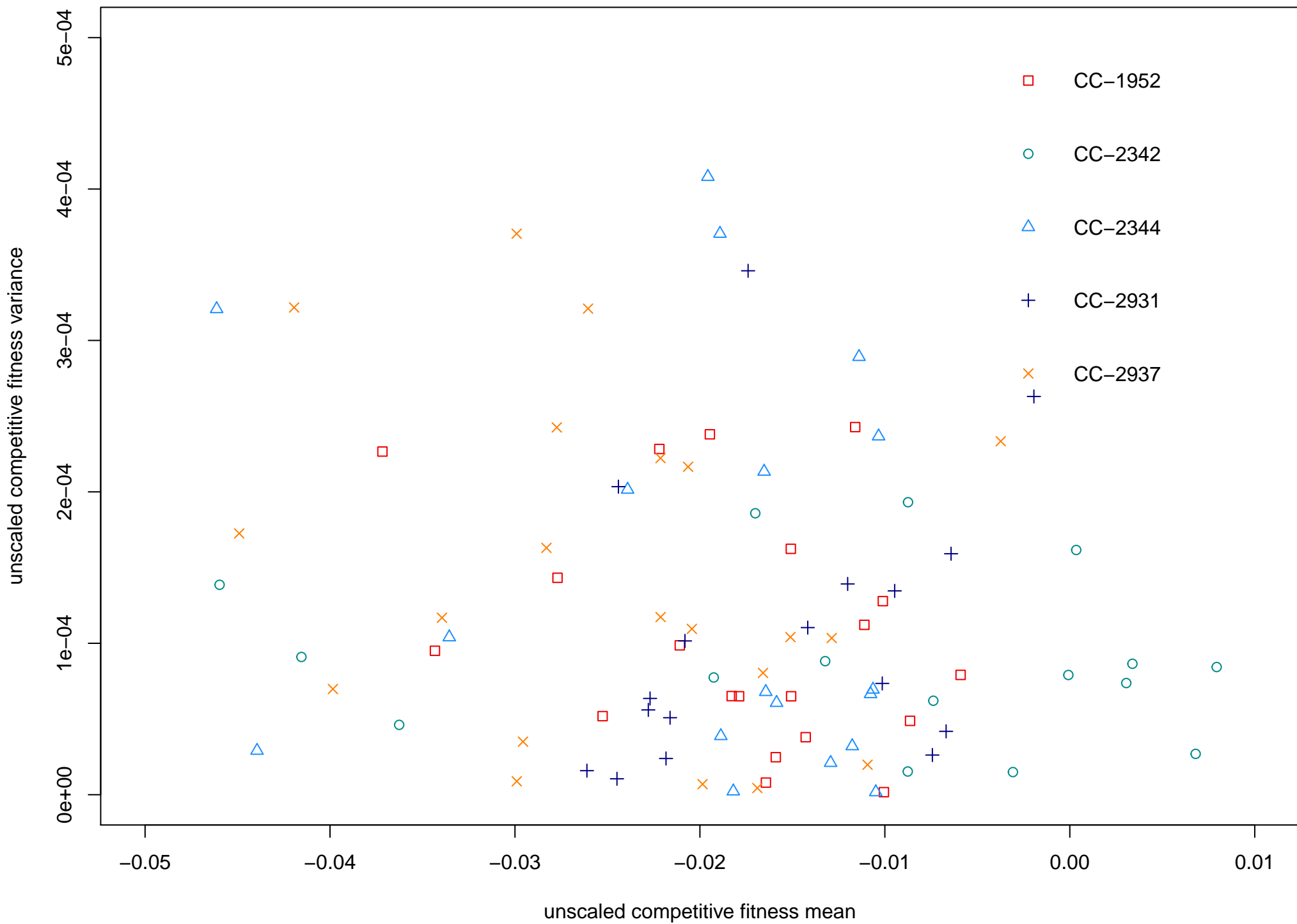

Supplement: Supplementary file 5 — Figure S5. Mean and variance of unscaled competitive fitness of each MA line. We did not detect a relationship between the mean and the variance of unscaled competitive fitness. [file EVO-71-2918-s005.pdf]
