# Supplementary material for: Fitness change in relation to mutation number in spontaneous mutation accumulation lines of Chlamydomonas reinhardtii
Source: Evolution. 2017 Oct 26;71(12):2918–29. doi: 10.1111/evo.13360 (PMC5765464; doi:10.1111/evo.13360)
Supplement: Supplementary file 8 — Supplemental material [file EVO-71-2918-s008.docx]

**Supplemental material**

1. Calculation of selective effects scaled by generation time

Competitive fitness can be scaled by ancestral generation time as described previously (Chevin, 2011, Kraemer *et al.*, 2015) to obtain fitness measures that reflect the expected change in frequency of a genotype per generation (*s_τMA_*):

*s_τMA_* = ln(2)*w_MA_/r_ANC_.* (S1)

Here, ln(2)/*r_ANC_* represents the ancestral generation time under the same environmental conditions as the MA line. To obtain comparable measures for the ancestors (*s_τANC_*), their competitive growth rates were scaled by their own generation time:

*s_τANC_* = ln(2)*w_ANC_/r_ANC_.* (S2)

In all cases, scaling by ancestral generation time was performed with ancestral growth rates obtained from the same assay plate in order to account for environment-induced variation in growth. The scaled selective effect for each MA line (*s*_τ_) was calculated as the difference between the scaled competitive fitnesses of the ma line (*s_τMA_*) and its ancestor (*s_τANC_*).

*s_τ_ = s_τANC_.- s_τMA_* (S3)

Relative fitness was then calculated as 1-*s_τ_*.

2. Equations for one and three categories of mutational effects

For *c* = 1 the likelihood equation is:

*L_i_* = ϕ (X_i_, *μ*, *V_E_*), (S4)

For *c = 3* the likelihood equation is:

$L_{i}= \sum_{j=0}^{n_{i}} \sum_{k=0}^{n_{i}-j} \sum_{l=0}^{n_{i}-j-k} f\left( 3,\mathbf{p}=\left[ p_{1},p_{2},1-p_{1}-p_{2} \right], \mathbf{n}=\left[ j,k,l \right] \right)\Phi\left( X_{i}-ks_{2}-ls_{\tau3},\mu,V_{E} \right).$(S5)
