# Supplementary material for: Fitness change in relation to mutation number in spontaneous mutation accumulation lines of Chlamydomonas reinhardtii
Source: Evolution. 2017 Oct 26;71(12):2918–29. doi: 10.1111/evo.13360 (PMC5765464; doi:10.1111/evo.13360)
Supplement: Supplementary file 9 — supplemental_statistics [file EVO-71-2918-s009.pdf]

supplemental_statistics

intergenic - indpendent of all others genic - includes everything that is not intergenic exonic - includes all genic expect intron UTR3 - within genic and exonic utr5 - within genic and exonic intron - within genic excluding exonic CDS - within exonic excluding UTR3 and UTR5

```
## Loading required package: Matrix

##
## Attaching package: 'lmerTest'

## The following object is masked from 'package:lme4':
##
##     lmer

## The following object is masked from 'package:stats':
##
##     step

##
## Attaching package: 'nlme'

## The following object is masked from 'package:lme4':
##
##     lmList
```

```
##Models with relative fitness as the response variable##
fit<-lmer(rel2~type+(1|anc),data0)
summary(fit)
```

```
## Linear mixed model fit by REML t-tests use Satterthwaite approximations
##   to degrees of freedom [lmerMod]
## Formula: rel2 ~ type + (1 | anc)
##    Data: data0
##
## REML criterion at convergence: -1695.7
##
## Scaled residuals:
##     Min      1Q  Median      3Q     Max
## -4.2956 -0.6096  0.0662  0.5042  3.8353
##
## Random effects:
##  Groups   Name        Variance  Std.Dev.
##  anc      (Intercept) 2.059e-05 0.004538
##  Residual             1.120e-04 0.010582
## Number of obs: 276, groups:  anc, 5
##
## Fixed effects:
##               Estimate Std. Error         df t value Pr(>|t|)
## (Intercept)   0.999753   0.002299   5.490000 434.809 1.17e-13 ***
## typema       -0.004622   0.001338 270.130000  -3.454 0.000641 ***
## ---
## Signif. codes:  0 '***' 0.001 '**' 0.01 '*' 0.05 '.' 0.1 ' ' 1
##
## Correlation of Fixed Effects:
##        (Intr)
```

```
## typema -0.379
```

```r
fit1<-lmer(rel2~total+(1|anc),data0)
summary(fit1)
```

```
## Linear mixed model fit by REML t-tests use Satterthwaite approximations
##   to degrees of freedom [lmerMod]
## Formula: rel2 ~ total + (1 | anc)
##    Data: data0
##
## REML criterion at convergence: -1682.6
##
## Scaled residuals:
##     Min      1Q  Median      3Q     Max
## -4.5797 -0.5341  0.1380  0.5026  3.7115
##
## Random effects:
##  Groups   Name        Variance  Std.Dev.
##  anc      (Intercept) 1.554e-05 0.003943
##  Residual             1.142e-04 0.010688
## Number of obs: 276, groups:  anc, 5
##
## Fixed effects:
##               Estimate Std. Error         df t value Pr(>|t|)
## (Intercept)  9.987e-01  2.003e-03  5.130e+00 498.527 3.25e-13 ***
## total       -4.868e-05  1.785e-05  2.739e+02  -2.727   0.0068 **
## ---
## Signif. codes:  0 '***' 0.001 '**' 0.01 '*' 0.05 '.' 0.1 ' ' 1
##
## Correlation of Fixed Effects:
##       (Intr)
## total -0.348
```

```r
AIC(fit1)
```

```
## [1] -1674.616
```

```r
fit2<-lmer(rel2~SNP+indel+(1|anc),data0)
summary(fit2)
```

```
## Linear mixed model fit by REML t-tests use Satterthwaite approximations
##   to degrees of freedom [lmerMod]
## Formula: rel2 ~ SNP + indel + (1 | anc)
##    Data: data0
##
## REML criterion at convergence: -1667.3
##
## Scaled residuals:
##     Min      1Q  Median      3Q     Max
## -4.5205 -0.5312  0.1298  0.5174  3.7286
##
## Random effects:
##  Groups   Name        Variance  Std.Dev.
##  anc      (Intercept) 1.689e-05 0.004109
##  Residual             1.141e-04 0.010681
## Number of obs: 276, groups:  anc, 5
```

```
##
## Fixed effects:
##               Estimate Std. Error         df t value Pr(>|t|)    
## (Intercept)  9.987e-01  2.069e-03  5.000e+00 482.651 7.26e-13 ***
## SNP         -2.879e-05  2.594e-05  2.730e+02  -1.110    0.268    
## indel       -1.409e-04  9.011e-05  2.714e+02  -1.563    0.119    
## ---
## Signif. codes:  0 '***' 0.001 '**' 0.01 '*' 0.05 '.' 0.1 ' ' 1
##
## Correlation of Fixed Effects:
##       (Intr) SNP   
## SNP   -0.229       
## indel -0.071 -0.575
```

```
AIC(fit2)
```

```
## [1] -1657.255
```

```
fit3<-lmer(rel2~nonsynonymous+synonymous+(1|anc),data0)
summary(fit3)
```

```
## Linear mixed model fit by REML t-tests use Satterthwaite approximations
##   to degrees of freedom [lmerMod]
## Formula: rel2 ~ nonsynonymous + synonymous + (1 | anc)
##    Data: data0
##
## REML criterion at convergence: -1675.2
##
## Scaled residuals: 
##     Min      1Q  Median      3Q     Max 
## -4.6521 -0.5507  0.1088  0.5120  3.8165 
##
## Random effects:
##  Groups   Name        Variance  Std.Dev.
##  anc      (Intercept) 1.453e-05 0.003812
##  Residual             1.129e-04 0.010626
## Number of obs: 276, groups:  anc, 5
##
## Fixed effects:
##                 Estimate Std. Error         df t value Pr(>|t|)    
## (Intercept)    9.991e-01  1.946e-03  5.140e+00 513.444 2.57e-13 ***
## nonsynonymous -1.531e-04  1.242e-04  2.725e+02  -1.232    0.219    
## synonymous    -2.586e-04  2.367e-04  2.717e+02  -1.093    0.276    
## ---
## Signif. codes:  0 '***' 0.001 '**' 0.01 '*' 0.05 '.' 0.1 ' ' 1
##
## Correlation of Fixed Effects:
##             (Intr) nnsynn
## nonsynonyms -0.130       
## synonymous  -0.105 -0.775
```

```
fit4<-lmer(rel2~intergenic+intronic+CDS+utr+(1|anc),data0)
summary(fit4)
```

```
## Linear mixed model fit by REML t-tests use Satterthwaite approximations
##   to degrees of freedom [lmerMod]
```

```
## Formula: rel2 ~ intergenic + intronic + CDS + utr + (1 | anc)
##    Data: data0
##
## REML criterion at convergence: -1650.6
##
## Scaled residuals:
##     Min      1Q  Median      3Q     Max
## -4.7408 -0.5655  0.0981  0.4959  4.1134
##
## Random effects:
##  Groups   Name        Variance  Std.Dev.
##  anc      (Intercept) 1.261e-05 0.003552
##  Residual             1.108e-04 0.010526
## Number of obs: 276, groups:  anc, 5
##
## Fixed effects:
##               Estimate Std. Error         df t value Pr(>|t|)
## (Intercept)  9.989e-01  1.846e-03  5.160e+00 541.051 1.81e-13 ***
## intergenic   1.906e-04  3.124e-04  2.705e+02   0.610 0.542231
## intronic     2.841e-04  1.361e-04  2.707e+02   2.088 0.037761 *
## CDS         -4.752e-04  1.284e-04  2.709e+02  -3.702 0.000259 ***
## utr         -1.926e-05  1.690e-04  2.646e+02  -0.114 0.909339
## ---
## Signif. codes:  0 '***' 0.001 '**' 0.01 '*' 0.05 '.' 0.1 ' ' 1
##
## Correlation of Fixed Effects:
##            (Intr) intrgn intrnc CDS
## intergenic -0.054
## intronic   -0.014 -0.262
## CDS        -0.091 -0.122 -0.534
## utr        -0.010 -0.249 -0.360 -0.314
```

```
AIC(fit4)
```

```
## [1] -1636.566
```

```
fit5<-lmer(rel2~exonic+intronic+intergenic+(1|anc),data0)
summary(fit5)
```

```
## Linear mixed model fit by REML t-tests use Satterthwaite approximations
##   to degrees of freedom [lmerMod]
## Formula: rel2 ~ exonic + intronic + intergenic + (1 | anc)
##    Data: data0
##
## REML criterion at convergence: -1661.9
##
## Scaled residuals:
##     Min      1Q  Median      3Q     Max
## -4.7368 -0.5651  0.1256  0.5152  4.1741
##
## Random effects:
##  Groups   Name        Variance  Std.Dev.
##  anc      (Intercept) 1.057e-05 0.003252
##  Residual             1.121e-04 0.010586
## Number of obs: 276, groups:  anc, 5
```

```
##
## Fixed effects:
##               Estimate Std. Error        df t value Pr(>|t|)
## (Intercept)  9.988e-01  1.733e-03  5.450e+00 576.432 2.98e-14 ***
## exonic      -2.988e-04  8.558e-05  2.670e+02  -3.492 0.000561 ***
## intronic     2.796e-04  1.367e-04  2.720e+02   2.046 0.041699 *
## intergenic   2.622e-04  3.124e-04  2.712e+02   0.839 0.402054
## ---
## Signif. codes:  0 '***' 0.001 '**' 0.01 '*' 0.05 '.' 0.1 ' ' 1
##
## Correlation of Fixed Effects:
##            (Intr) exonic intrnc
## exonic     -0.095
## intronic   -0.018 -0.770
## intergenic -0.054 -0.311 -0.258
```

```
AIC(fit5)
```

```
## [1] -1649.935
```

Mutational effects and competitive fitness

```
##Models with competitive fitness as the response variable##
fit<-lmer(w_ma~type+(1|anc),data0)
summary(fit)
```

```
## Linear mixed model fit by REML t-tests use Satterthwaite approximations
##   to degrees of freedom [lmerMod]
## Formula: w_ma ~ type + (1 | anc)
##    Data: data0
##
## REML criterion at convergence: -1555.5
##
## Scaled residuals:
##     Min      1Q  Median      3Q     Max
## -3.1986 -0.5400  0.0633  0.7303  2.5003
##
## Random effects:
##  Groups   Name        Variance  Std.Dev.
##  anc      (Intercept) 1.882e-05 0.004338
##  Residual             1.882e-04 0.013720
## Number of obs: 276, groups:  anc, 5
##
## Fixed effects:
##               Estimate Std. Error        df t value Pr(>|t|)
## (Intercept)  -0.012956   0.002393   6.610000  -5.413   0.0012 **
## typema       -0.007507   0.001735 270.120000  -4.327 2.13e-05 ***
## ---
## Signif. codes:  0 '***' 0.001 '**' 0.01 '*' 0.05 '.' 0.1 ' ' 1
##
## Correlation of Fixed Effects:
##        (Intr)
## typema -0.472
```

```
AIC(fit)
```

```
## [1] -1547.502
```

```r
fit1a<-lmer(w_ma~total+(1|anc),data0)
summary(fit1a)
```

```
## Linear mixed model fit by REML t-tests use Satterthwaite approximations
##   to degrees of freedom [lmerMod]
## Formula: w_ma ~ total + (1 | anc)
##    Data: data0
##
## REML criterion at convergence: -1533.8
##
## Scaled residuals:
##     Min      1Q  Median      3Q     Max
## -3.3737 -0.5931  0.1303  0.6644  2.2512
##
## Random effects:
##  Groups   Name        Variance  Std.Dev.
##  anc      (Intercept) 2.535e-05 0.005035
##  Residual             1.968e-04 0.014029
## Number of obs: 276, groups:  anc, 5
##
## Fixed effects:
##               Estimate Std. Error         df t value Pr(>|t|)
## (Intercept) -1.575e-02  2.574e-03  5.100e+00  -6.120  0.00157 **
## total       -5.326e-05  2.342e-05  2.738e+02  -2.274  0.02375 *
## ---
## Signif. codes:  0 '***' 0.001 '**' 0.01 '*' 0.05 '.' 0.1 ' ' 1
##
## Correlation of Fixed Effects:
##       (Intr)
## total -0.355
```

```r
AIC(fit1a)
```

```
## [1] -1525.755
```

```r
fit2a<-lmer(w_ma~SNP+indel+(1|anc),data0)
summary(fit2a)
```

```
## Linear mixed model fit by REML t-tests use Satterthwaite approximations
##   to degrees of freedom [lmerMod]
## Formula: w_ma ~ SNP + indel + (1 | anc)
##    Data: data0
##
## REML criterion at convergence: -1518
##
## Scaled residuals:
##     Min      1Q  Median      3Q     Max
## -3.3701 -0.5969  0.1200  0.6836  2.2311
##
## Random effects:
##  Groups   Name        Variance  Std.Dev.
##  anc      (Intercept) 2.462e-05 0.004962
##  Residual             1.975e-04 0.014053
## Number of obs: 276, groups:  anc, 5
##
```

```
## Fixed effects:
##               Estimate Std. Error         df t value Pr(>|t|)
## (Intercept) -1.575e-02  2.547e-03  5.080e+00  -6.186  0.00152 **
## SNP         -4.239e-05  3.408e-05  2.727e+02  -1.244  0.21467
## indel       -1.035e-04  1.183e-04  2.697e+02  -0.875  0.38236
## ---
## Signif. codes:  0 '***' 0.001 '**' 0.01 '*' 0.05 '.' 0.1 ' ' 1
##
## Correlation of Fixed Effects:
##       (Intr) SNP
## SNP   -0.244
## indel -0.076 -0.575
```

```
AIC(fit2a)
```

```
## [1] -1508.041
```

```
fit3a<-lmer(w_ma~nonsynonymous+synonymous+(1|anc),data0)
summary(fit3a)
```

```
## Linear mixed model fit by REML t-tests use Satterthwaite approximations
##   to degrees of freedom [lmerMod]
## Formula: w_ma ~ nonsynonymous + synonymous + (1 | anc)
##    Data: data0
##
## REML criterion at convergence: -1526.9
##
## Scaled residuals:
##     Min      1Q  Median      3Q     Max
## -3.2411 -0.6198  0.1403  0.6845  2.2597
##
## Random effects:
##  Groups   Name        Variance  Std.Dev.
##  anc      (Intercept) 2.767e-05 0.00526
##  Residual             1.941e-04 0.01393
## Number of obs: 276, groups:  anc, 5
##
## Fixed effects:
##                 Estimate Std. Error         df t value Pr(>|t|)
## (Intercept)   -1.504e-02  2.655e-03  4.970e+00  -5.665  0.00243 **
## nonsynonymous -2.471e-04  1.629e-04  2.723e+02  -1.517  0.13049
## synonymous    -1.721e-04  3.104e-04  2.714e+02  -0.554  0.57972
## ---
## Signif. codes:  0 '***' 0.001 '**' 0.01 '*' 0.05 '.' 0.1 ' ' 1
##
## Correlation of Fixed Effects:
##             (Intr) nnsynn
## nonsynonyms -0.126
## synonymous  -0.101 -0.775
```

```
AIC(fit3a)
```

```
## [1] -1516.858
```

```
fit4a<-lmer(w_ma~intergenic+intronic+CDS+utr+(1|anc),data0)
summary(fit4a)
```

```
## Linear mixed model fit by REML t-tests use Satterthwaite approximations
##   to degrees of freedom [lmerMod]
## Formula: w_ma ~ intergenic + intronic + CDS + utr + (1 | anc)
##    Data: data0
##
## REML criterion at convergence: -1504.5
##
## Scaled residuals:
##     Min      1Q  Median      3Q     Max
## -2.9741 -0.5930  0.1359  0.6943  2.3844
##
## Random effects:
##  Groups   Name        Variance  Std.Dev.
##  anc      (Intercept) 3.052e-05 0.005524
##  Residual             1.891e-04 0.013750
## Number of obs: 276, groups:  anc, 5
##
## Fixed effects:
##               Estimate Std. Error        df t value Pr(>|t|)
## (Intercept) -1.538e-02  2.761e-03  4.740e+00  -5.570 0.003038 **
## intergenic   2.338e-04  4.085e-04  2.699e+02   0.572 0.567555
## intronic     3.898e-04  1.780e-04  2.701e+02   2.190 0.029398 *
## CDS         -6.497e-04  1.679e-04  2.704e+02  -3.869 0.000137 ***
## utr          3.784e-05  2.218e-04  2.691e+02   0.171 0.864645
## ---
## Signif. codes:  0 '***' 0.001 '**' 0.01 '*' 0.05 '.' 0.1 ' ' 1
##
## Correlation of Fixed Effects:
##            (Intr) intrgn intrnc CDS
## intergenic -0.047
## intronic   -0.011 -0.261
## CDS        -0.080 -0.121 -0.534
## utr        -0.010 -0.252 -0.360 -0.312
```

```r
AIC(fit4a)
```

```
## [1] -1490.545
```

```r
fit5a<-lmer(w_ma~exonic+intronic+intergenic+(1|anc),data0)
summary(fit5a)
```

```
## Linear mixed model fit by REML t-tests use Satterthwaite approximations
##   to degrees of freedom [lmerMod]
## Formula: w_ma ~ exonic + intronic + intergenic + (1 | anc)
##    Data: data0
##
## REML criterion at convergence: -1514.1
##
## Scaled residuals:
##      Min       1Q   Median       3Q      Max
## -2.95638 -0.59818  0.09337  0.65332  2.42064
##
## Random effects:
##  Groups   Name        Variance  Std.Dev.
##  anc      (Intercept) 0.0000369 0.006075
```

```
##  Residual             0.0001912 0.013827
## Number of obs: 276, groups:  anc, 5
##
## Fixed effects:
##               Estimate Std. Error         df t value Pr(>|t|)
## (Intercept) -1.556e-02  2.985e-03  4.670e+00  -5.213 0.004190 **
## exonic      -3.819e-04  1.126e-04  2.719e+02  -3.392 0.000798 ***
## intronic     3.799e-04  1.790e-04  2.708e+02   2.122 0.034754 *
## intergenic   3.426e-04  4.088e-04  2.699e+02   0.838 0.402712
## ---
## Signif. codes:  0 '***' 0.001 '**' 0.01 '*' 0.05 '.' 0.1 ' ' 1
##
## Correlation of Fixed Effects:
##            (Intr) exonic intrnc
## exonic     -0.076
## intronic   -0.011 -0.770
## intergenic -0.040 -0.313 -0.256
```

```
AIC(fit5a)
```

```
## [1] -1502.125
```

```
##Mutational effects and stress, relative fitness and competitive fitness##
datan$treatment<-as.factor(datan$treatment)
fit<-lmer(rel2~treatment*type+(1|anc),datan)
summary(fit)
```

```
## Linear mixed model fit by REML t-tests use Satterthwaite approximations
##   to degrees of freedom [lmerMod]
## Formula: rel2 ~ treatment * type + (1 | anc)
##    Data: datan
##
## REML criterion at convergence: -3136
##
## Scaled residuals:
##     Min      1Q  Median      3Q     Max
## -3.9114 -0.5268 -0.0132  0.4701  5.9191
##
## Random effects:
##  Groups   Name        Variance  Std.Dev.
##  anc      (Intercept) 1.739e-05 0.00417
##  Residual             1.376e-04 0.01173
## Number of obs: 527, groups:  anc, 5
##
## Fixed effects:
##                      Estimate Std. Error         df t value Pr(>|t|)
## (Intercept)         9.998e-01  2.216e-03  6.900e+00 451.087  < 2e-16 ***
## treatment25         1.374e-04  1.722e-03  5.192e+02   0.080  0.93641
## typema             -4.616e-03  1.483e-03  5.191e+02  -3.113  0.00195 **
## treatment25:typema  3.627e-03  2.141e-03  5.191e+02   1.694  0.09079 .
## ---
## Signif. codes:  0 '***' 0.001 '**' 0.01 '*' 0.05 '.' 0.1 ' ' 1
##
## Correlation of Fixed Effects:
##             (Intr) trtm25 typema
## treatment25 -0.376
```

```
## typema      -0.436  0.561
## trtmnt25:ty  0.302 -0.804 -0.692
```

```r
AIC(fit)
```

```
## [1] -3124.002
```

```r
fita<-lmer(w_ma~treatment*type+(1|anc),datan)
summary(fita)
```

```
## Linear mixed model fit by REML t-tests use Satterthwaite approximations
##   to degrees of freedom [lmerMod]
## Formula: w_ma ~ treatment * type + (1 | anc)
##    Data: datan
##
## REML criterion at convergence: -2802.3
##
## Scaled residuals:
##     Min      1Q  Median      3Q     Max
## -3.4070 -0.5707  0.0583  0.6893  2.9823
##
## Random effects:
##  Groups   Name        Variance  Std.Dev.
##  anc      (Intercept) 2.475e-05 0.004975
##  Residual             2.609e-04 0.016152
## Number of obs: 527, groups:  anc, 5
##
## Fixed effects:
##                       Estimate Std. Error         df t value Pr(>|t|)
## (Intercept)         -1.288e-02  2.770e-03  8.000e+00  -4.652 0.001668 **
## treatment25          4.620e-03  2.371e-03  5.192e+02   1.949 0.051877 .
## typema              -7.479e-03  2.042e-03  5.191e+02  -3.663 0.000275 ***
## treatment25:typema  1.621e-05  2.948e-03  5.191e+02   0.006 0.995613
## ---
## Signif. codes:  0 '***' 0.001 '**' 0.01 '*' 0.05 '.' 0.1 ' ' 1
##
## Correlation of Fixed Effects:
##             (Intr) trtm25 typema
## treatment25 -0.414
## typema      -0.480  0.561
## trtmnt25:ty  0.333 -0.804 -0.692
```

```r
AIC(fita)
```

```
## [1] -2790.348
```

```r
fit6<-lmer(rel2~total*treatment+(1|anc),datan)
summary(fit6)
```

```
## Linear mixed model fit by REML t-tests use Satterthwaite approximations
##   to degrees of freedom [lmerMod]
## Formula: rel2 ~ total * treatment + (1 | anc)
##    Data: datan
##
## REML criterion at convergence: -3115.4
##
## Scaled residuals:
```

```
##     Min      1Q  Median      3Q     Max 
## -4.1506 -0.5261  0.0351  0.4552  5.8785 
## 
## Random effects:
##  Groups   Name        Variance  Std.Dev.
##  anc      (Intercept) 1.354e-05 0.00368 
##  Residual             1.386e-04 0.01177 
## Number of obs: 527, groups:  anc, 5
## 
## Fixed effects:
##                     Estimate Std. Error         df t value Pr(>|t|)    
## (Intercept)        9.986e-01  1.939e-03  6.300e+00 514.903 6.66e-16 ***
## total             -4.568e-05  1.915e-05  5.231e+02  -2.386   0.0174 *  
## treatment25        1.711e-03  1.448e-03  5.194e+02   1.181   0.2380    
## total:treatment25  2.055e-05  2.652e-05  5.191e+02   0.775   0.4387    
## ---
## Signif. codes:  0 '***' 0.001 '**' 0.01 '*' 0.05 '.' 0.1 ' ' 1
## 
## Correlation of Fixed Effects:
##             (Intr) total  trtm25
## total       -0.381              
## treatment25 -0.360  0.480       
## ttl:trtmn25  0.255 -0.679 -0.704
```

```
AIC(fit6)
```

```
## [1] -3103.42
```

```
fit6a<-lmer(w_ma~total*treatment+(1|anc),datan)
summary(fit6a)
```

```
## Linear mixed model fit by REML t-tests use Satterthwaite approximations
##   to degrees of freedom [lmerMod]
## Formula: w_ma ~ total * treatment + (1 | anc)
##    Data: datan
## 
## REML criterion at convergence: -2774.2
## 
## Scaled residuals: 
##     Min      1Q  Median      3Q     Max 
## -3.4735 -0.5532  0.1107  0.6709  2.8995 
## 
## Random effects:
##  Groups   Name        Variance  Std.Dev.
##  anc      (Intercept) 3.635e-05 0.006029
##  Residual             2.656e-04 0.016298
## Number of obs: 527, groups:  anc, 5
## 
## Fixed effects:
##                     Estimate Std. Error         df t value Pr(>|t|)   
## (Intercept)       -1.514e-02  3.048e-03  5.600e+00  -4.969  0.00302 **
## total             -6.614e-05  2.653e-05  5.227e+02  -2.493  0.01296 * 
## treatment25        5.337e-03  2.005e-03  5.193e+02   2.662  0.00801 **
## total:treatment25 -1.690e-05  3.671e-05  5.191e+02  -0.460  0.64536   
## ---
```

```
## Signif. codes:  0 '***' 0.001 '**' 0.01 '*' 0.05 '.' 0.1 ' ' 1
##
## Correlation of Fixed Effects:
##              (Intr) total  trtm25
## total        -0.336
## treatment25 -0.317  0.480
## ttl:trtmn25  0.225 -0.678 -0.704
```

```
AIC(fit6a)
```

```
## [1] -2762.16
```

```
fit7<-lmer(rel2~SNP*treatment+indel*treatment+(1|anc),datan)
summary(fit7)
```

```
## Linear mixed model fit by REML t-tests use Satterthwaite approximations
##   to degrees of freedom [lmerMod]
## Formula: rel2 ~ SNP * treatment + indel * treatment + (1 | anc)
##    Data: datan
##
## REML criterion at convergence: -3084.7
##
## Scaled residuals:
##     Min      1Q  Median      3Q     Max
## -4.1550 -0.5009  0.0317  0.4497  5.8273
##
## Random effects:
##  Groups   Name        Variance  Std.Dev.
##  anc      (Intercept) 1.333e-05 0.003651
##  Residual             1.387e-04 0.011777
## Number of obs: 527, groups:  anc, 5
##
## Fixed effects:
##                      Estimate Std. Error         df t value Pr(>|t|)
## (Intercept)         9.986e-01  1.928e-03  6.400e+00 517.858 6.66e-16 ***
## SNP                -3.356e-05  2.784e-05  5.211e+02  -1.205    0.229
## treatment25         1.690e-03  1.449e-03  5.174e+02   1.166    0.244
## indel              -1.010e-04  9.567e-05  5.205e+02  -1.056    0.292
## SNP:treatment25    -1.622e-05  3.843e-05  5.171e+02  -0.422    0.673
## treatment25:indel   1.911e-04  1.312e-04  5.172e+02   1.456    0.146
## ---
## Signif. codes:  0 '***' 0.001 '**' 0.01 '*' 0.05 '.' 0.1 ' ' 1
##
## Correlation of Fixed Effects:
##              (Intr) SNP    trtm25 indel  SNP:25
## SNP          -0.262
## treatment25 -0.362  0.328
## indel        -0.080 -0.574  0.098
## SNP:trtmn25  0.176 -0.681 -0.479  0.385
## trtmnt25:nd  0.054  0.385 -0.152 -0.668 -0.569
```

```
AIC(fit7)
```

```
## [1] -3068.731
```

```
fit7a<-lmer(w_ma~SNP*treatment+indel*treatment+(1|anc),datan)
summary(fit7a)
```

```
## Linear mixed model fit by REML t-tests use Satterthwaite approximations
##   to degrees of freedom [lmerMod]
## Formula: w_ma ~ SNP * treatment + indel * treatment + (1 | anc)
##    Data: datan
##
## REML criterion at convergence: -2744.8
##
## Scaled residuals:
##     Min      1Q  Median      3Q     Max
## -3.4503 -0.5482  0.1130  0.6522  2.9541
##
## Random effects:
##  Groups   Name        Variance  Std.Dev.
##  anc      (Intercept) 3.764e-05 0.006135
##  Residual             2.656e-04 0.016297
## Number of obs: 527, groups:  anc, 5
##
## Fixed effects:
##                      Estimate Std. Error         df t value Pr(>|t|)
## (Intercept)        -1.514e-02  3.090e-03  5.600e+00  -4.899  0.00335 **
## SNP                -5.663e-05  3.857e-05  5.206e+02  -1.468  0.14268
## treatment25         5.306e-03  2.005e-03  5.173e+02   2.647  0.00837 **
## indel              -1.091e-04  1.326e-04  5.210e+02  -0.823  0.41089
## SNP:treatment25    -6.350e-05  5.318e-05  5.170e+02  -1.194  0.23301
## treatment25:indel  1.997e-04  1.815e-04  5.171e+02   1.100  0.27188
## ---
## Signif. codes:  0 '***' 0.001 '**' 0.01 '*' 0.05 '.' 0.1 ' ' 1
##
## Correlation of Fixed Effects:
##             (Intr) SNP    trtm25 indel  SNP:25
## SNP         -0.226
## treatment25 -0.313  0.328
## indel       -0.069 -0.574  0.098
## SNP:trtmn25  0.152 -0.680 -0.479  0.385
## trtmnt25:nd  0.046  0.385 -0.152 -0.667 -0.569
```

```
AIC(fit7a)
```

```
## [1] -2728.757
```

```
fit8<-lmer(rel2~nonsynonymous*treatment+synonymous*treatment+(1|anc),datan)
summary(fit8)
```

```
## Linear mixed model fit by REML t-tests use Satterthwaite approximations
##   to degrees of freedom [lmerMod]
## Formula: rel2 ~ nonsynonymous * treatment + synonymous * treatment + (1 |
##     anc)
##    Data: datan
##
## REML criterion at convergence: -3103.8
##
## Scaled residuals:
##     Min      1Q  Median      3Q     Max
## -4.2516 -0.5034  0.0107  0.4700  5.8186
##
```

```
## Random effects:
##  Groups   Name        Variance  Std.Dev.
##  anc      (Intercept) 0.0000118 0.003436
##  Residual             0.0001362 0.011671
## Number of obs: 527, groups:  anc, 5
##
## Fixed effects:
##                            Estimate Std. Error         df t value
## (Intercept)               9.991e-01  1.838e-03  6.600e+00 543.519
## nonsynonymous            -1.412e-04  1.346e-04  5.201e+02  -1.049
## treatment25               1.590e-03  1.423e-03  5.174e+02   1.117
## synonymous               -2.697e-04  2.576e-04  5.194e+02  -1.047
## nonsynonymous:treatment25 -2.717e-04  1.899e-04  5.171e+02  -1.431
## treatment25:synonymous     7.529e-04  3.692e-04  5.171e+02   2.039
##                          Pr(>|t|)
## (Intercept)                <2e-16 ***
## nonsynonymous              0.2948
## treatment25                0.2644
## synonymous                 0.2956
## nonsynonymous:treatment25   0.1531
## treatment25:synonymous     0.0419 *
## ---
## Signif. codes:  0 '***' 0.001 '**' 0.01 '*' 0.05 '.' 0.1 ' ' 1
##
## Correlation of Fixed Effects:
##             (Intr) nnsynn trtm25 synnym nns:25
## nonsynonyms -0.141
## treatment25 -0.374  0.167
## synonymous  -0.119 -0.782  0.148
## nnsynnym:25  0.090 -0.686 -0.234  0.544
## trtmnt25:sy  0.079  0.536 -0.215 -0.682 -0.793
```

```r
AIC(fit8)
```

```
## [1] -3087.783
```

```r
fit8a<-lmer(w_ma~nonsynonymous*treatment+synonymous*treatment+(1|anc),datan)
summary(fit8a)
```

```
## Linear mixed model fit by REML t-tests use Satterthwaite approximations
##   to degrees of freedom [lmerMod]
## Formula: w_ma ~ nonsynonymous * treatment + synonymous * treatment + (1 |
##     anc)
##    Data: datan
##
## REML criterion at convergence: -2760.7
##
## Scaled residuals:
##     Min      1Q  Median      3Q     Max
## -3.5390 -0.5785  0.1215  0.6873  2.9022
##
## Random effects:
##  Groups   Name        Variance  Std.Dev.
##  anc      (Intercept) 3.998e-05 0.006323
##  Residual             2.621e-04 0.016190
```

```
## Number of obs: 527, groups:  anc, 5
##
## Fixed effects:
##                              Estimate Std. Error         df t value
## (Intercept)                -1.443e-02  3.156e-03  5.400e+00  -4.571
## nonsynonymous              -2.940e-04  1.869e-04  5.191e+02  -1.574
## treatment25                 4.962e-03  1.974e-03  5.172e+02   2.513
## synonymous                 -2.008e-04  3.576e-04  5.185e+02  -0.562
## nonsynonymous:treatment25 -2.110e-04  2.635e-04  5.170e+02  -0.801
## treatment25:synonymous      3.470e-04  5.122e-04  5.170e+02   0.678
##                            Pr(>|t|)
## (Intercept)                 0.00489 **
## nonsynonymous               0.11619
## treatment25                 0.01227 *
## synonymous                  0.57464
## nonsynonymous:treatment25   0.42346
## treatment25:synonymous      0.49837
## ---
## Signif. codes:  0 '***' 0.001 '**' 0.01 '*' 0.05 '.' 0.1 ' ' 1
##
## Correlation of Fixed Effects:
##             (Intr) nnsynn trtm25 synnym nns:25
## nonsynonyms -0.114
## treatment25 -0.302  0.167
## synonymous  -0.096 -0.781  0.148
## nnsynnym:25  0.073 -0.686 -0.234  0.544
## trtmnt25:sy  0.064  0.535 -0.215 -0.681 -0.793
```

```
AIC(fit8a)
```

```
## [1] -2744.692
```

```
fit9<-lmer(rel2~intergenic*treatment+intronic*treatment+CDS*treatment+utr*treatment+(1|anc),datan)
summary(fit9)
```

```
## Linear mixed model fit by REML t-tests use Satterthwaite approximations
##   to degrees of freedom [lmerMod]
## Formula: rel2 ~ intergenic * treatment + intronic * treatment + CDS *
##     treatment + utr * treatment + (1 | anc)
##    Data: datan
##
## REML criterion at convergence: -3050
##
## Scaled residuals:
##     Min      1Q  Median      3Q     Max
## -4.3017 -0.5331  0.0177  0.4521  5.6908
##
## Random effects:
##  Groups   Name        Variance  Std.Dev.
##  anc      (Intercept) 1.097e-05 0.003312
##  Residual             1.348e-04 0.011609
## Number of obs: 527, groups:  anc, 5
##
## Fixed effects:
##                           Estimate Std. Error         df t value Pr(>|t|)
```

```
## (Intercept)             9.988e-01  1.798e-03  6.600e+00 555.588  < 2e-16
## intergenic              1.729e-04  3.410e-04  5.160e+02   0.507 0.612305
## treatment25             1.509e-03  1.440e-03  5.134e+02   1.048 0.295291
## intronic                2.979e-04  1.481e-04  5.164e+02   2.011 0.044820
## CDS                    -4.824e-04  1.394e-04  5.167e+02  -3.460 0.000585
## utr                    -9.381e-06  1.796e-04  5.143e+02  -0.052 0.958366
## intergenic:treatment25  6.586e-04  4.861e-04  5.131e+02   1.355 0.176016
## treatment25:intronic   -2.182e-04  2.093e-04  5.131e+02  -1.042 0.297787
## treatment25:CDS         6.138e-05  1.992e-04  5.131e+02   0.308 0.758057
## treatment25:utr         6.739e-05  2.468e-04  5.131e+02   0.273 0.784903
##
## (Intercept)            ***
## intergenic
## treatment25
## intronic               *
## CDS                    ***
## utr
## intergenic:treatment25
## treatment25:intronic
## treatment25:CDS
## treatment25:utr
## ---
## Signif. codes:  0 '***' 0.001 '**' 0.01 '*' 0.05 '.' 0.1 ' ' 1
##
## Correlation of Fixed Effects:
##              (Intr) intrgn trtm25 intrnc CDS    utr    int:25 trtmnt25:n
## intergenic  -0.068
## treatment25 -0.387  0.091
## intronic    -0.029 -0.272  0.051
## CDS         -0.093 -0.136  0.102 -0.527
## utr          0.005 -0.222 -0.026 -0.357 -0.324
## intrgnc:t25  0.051 -0.682 -0.126  0.193  0.107  0.122
## trtmnt25:nt  0.033  0.194 -0.084 -0.683  0.351  0.237 -0.285
## trtmn25:CDS  0.052  0.106 -0.131  0.348 -0.669  0.220 -0.153 -0.526
## trtmnt25:tr -0.016  0.128  0.033  0.244  0.226 -0.660 -0.195 -0.331
##             t25:CD
## intergenic
## treatment25
## intronic
## CDS
## utr
## intrgnc:t25
## trtmnt25:nt
## trtmn25:CDS
## trtmnt25:tr -0.352
```

```r
AIC(fit9)
```

```
## [1] -3026.008
```

```r
fit9a<-lmer(w_ma~intergenic*treatment+intronic*treatment+CDS*treatment+utr*treatment+(1|anc),datan)
summary(fit9a)
```

```
## Linear mixed model fit by REML t-tests use Satterthwaite approximations
##   to degrees of freedom [lmerMod]
```

```
## Formula: w_ma ~ intergenic * treatment + intronic * treatment + CDS *
##     treatment + utr * treatment + (1 | anc)
##    Data: datan
##
## REML criterion at convergence: -2710.2
##
## Scaled residuals:
##     Min      1Q  Median      3Q     Max
## -3.9601 -0.5728  0.1077  0.6679  3.0266
##
## Random effects:
##  Groups   Name        Variance  Std.Dev.
##  anc      (Intercept) 4.552e-05 0.006747
##  Residual             2.586e-04 0.016082
## Number of obs: 527, groups:  anc, 5
##
## Fixed effects:
##                         Estimate Std. Error         df t value Pr(>|t|)
## (Intercept)           -1.479e-02  3.332e-03  5.200e+00  -4.440  0.00617
## intergenic             3.029e-04  4.728e-04  5.147e+02   0.641  0.52202
## treatment25            5.007e-03  1.995e-03  5.132e+02   2.510  0.01240
## intronic               4.379e-04  2.054e-04  5.150e+02   2.132  0.03351
## CDS                   -6.880e-04  1.934e-04  5.153e+02  -3.557  0.00041
## utr                   -5.899e-05  2.497e-04  5.170e+02  -0.236  0.81334
## intergenic:treatment25  7.810e-04  6.734e-04  5.130e+02   1.160  0.24665
## treatment25:intronic   -4.535e-04  2.900e-04  5.130e+02  -1.564  0.11843
## treatment25:CDS         2.371e-04  2.759e-04  5.130e+02   0.859  0.39064
## treatment25:utr        -5.344e-05  3.419e-04  5.130e+02  -0.156  0.87584
##
## (Intercept)           **
## intergenic
## treatment25           *
## intronic              *
## CDS                   ***
## utr
## intergenic:treatment25
## treatment25:intronic
## treatment25:CDS
## treatment25:utr
## ---
## Signif. codes:  0 '***' 0.001 '**' 0.01 '*' 0.05 '.' 0.1 ' ' 1
##
## Correlation of Fixed Effects:
##             (Intr) intrgn trtm25 intrnc CDS    utr    int:25 trtmnt25:n
## intergenic  -0.050
## treatment25 -0.290  0.091
## intronic    -0.021 -0.271  0.050
## CDS         -0.070 -0.135  0.102 -0.528
## utr          0.002 -0.225 -0.026 -0.358 -0.323
## intrgnc:t25  0.038 -0.681 -0.126  0.193  0.107  0.121
## trtmnt25:nt  0.024  0.194 -0.084 -0.682  0.350  0.236 -0.285
## trtmn25:CDS  0.039  0.106 -0.131  0.347 -0.668  0.219 -0.153 -0.526
## trtmnt25:tr -0.012  0.127  0.033  0.244  0.226 -0.658 -0.195 -0.331
##             t25:CD
```

```
## intergenic
## treatment25
## intronic
## CDS
## utr
## intrgnc:t25
## trtmnt25:nt
## trtmn25:CDS
## trtmnt25:tr -0.352
```

```r
AIC(fit9a)
```

```
## [1] -2686.176
```

```r
fit10<-lmer(rel2~exonic*treatment+intronic*treatment+intergenic*treatment+(1|anc),datan)
summary(fit10)
```

```
## Linear mixed model fit by REML t-tests use Satterthwaite approximations
##   to degrees of freedom [lmerMod]
## Formula: rel2 ~ exonic * treatment + intronic * treatment + intergenic *
##     treatment + (1 | anc)
##    Data: datan
##
## REML criterion at convergence: -3073.6
##
## Scaled residuals:
##     Min      1Q  Median      3Q     Max
## -4.3060 -0.5120  0.0266  0.4446  5.6531
##
## Random effects:
##  Groups   Name        Variance  Std.Dev.
##  anc      (Intercept) 8.818e-06 0.00297
##  Residual             1.359e-04 0.01166
## Number of obs: 527, groups:  anc, 5
##
## Fixed effects:
##                          Estimate Std. Error         df t value Pr(>|t|)
## (Intercept)             9.987e-01  1.673e-03  7.400e+00 596.791  < 2e-16
## exonic                 -2.989e-04  9.141e-05  5.163e+02  -3.270  0.00115
## treatment25             1.556e-03  1.440e-03  5.155e+02   1.081  0.28027
## intronic                2.922e-04  1.486e-04  5.188e+02   1.966  0.04980
## intergenic              2.495e-04  3.413e-04  5.177e+02   0.731  0.46521
## exonic:treatment25      6.610e-05  1.260e-04  5.151e+02   0.525  0.59999
## treatment25:intronic   -2.277e-04  2.097e-04  5.151e+02  -1.086  0.27814
## treatment25:intergenic  6.688e-04  4.880e-04  5.151e+02   1.371  0.17113
##
## (Intercept)            ***
## exonic                 **
## treatment25
## intronic               *
## intergenic
## exonic:treatment25
## treatment25:intronic
## treatment25:intergenic
## ---
```

```
## Signif. codes:  0 '***' 0.001 '**' 0.01 '*' 0.05 '.' 0.1 ' ' 1
##
## Correlation of Fixed Effects:
##                 (Intr) exonic trtm25 intrnc intrgn exn:25 trtmnt25:ntrn
## exonic          -0.085
## treatment25     -0.415  0.070
## intronic        -0.034 -0.768  0.054
## intergenic      -0.069 -0.305  0.088 -0.269
## exnc:trtm25      0.038 -0.669 -0.092  0.524  0.205
## trtmnt25:ntrn    0.037  0.510 -0.091 -0.684  0.194 -0.761
## trtmnt25:ntrg    0.053  0.198 -0.122  0.191 -0.685 -0.306 -0.281
```

```r
AIC(fit10)
```

```
## [1] -3053.563
```

```r
fit10a<-lmer(w_ma~exonic*treatment+intronic*treatment+intergenic*treatment+(1|anc),datan)
summary(fit10a)
```

```
## Linear mixed model fit by REML t-tests use Satterthwaite approximations
##   to degrees of freedom [lmerMod]
## Formula: w_ma ~ exonic * treatment + intronic * treatment + intergenic *
##     treatment + (1 | anc)
##    Data: datan
##
## REML criterion at convergence: -2734.4
##
## Scaled residuals:
##     Min      1Q  Median      3Q     Max
## -3.9401 -0.5739  0.1027  0.6711  3.0341
##
## Random effects:
##  Groups   Name        Variance  Std.Dev.
##  anc      (Intercept) 5.029e-05 0.007091
##  Residual             2.593e-04 0.016102
## Number of obs: 527, groups:  anc, 5
##
## Fixed effects:
##                         Estimate Std. Error         df t value Pr(>|t|)
## (Intercept)           -1.500e-02  3.471e-03  5.100e+00  -4.323 0.007229
## exonic                -4.375e-04  1.268e-04  5.186e+02  -3.450 0.000606
## treatment25            5.165e-03  1.989e-03  5.152e+02   2.597 0.009669
## intronic               4.251e-04  2.056e-04  5.169e+02   2.068 0.039121
## intergenic             3.897e-04  4.719e-04  5.161e+02   0.826 0.409303
## exonic:treatment25     1.171e-04  1.740e-04  5.150e+02   0.673 0.501221
## treatment25:intronic  -4.528e-04  2.897e-04  5.150e+02  -1.563 0.118621
## treatment25:intergenic  7.719e-04  6.739e-04  5.150e+02   1.145 0.252584
##
## (Intercept)           **
## exonic                ***
## treatment25           **
## intronic              *
## intergenic
## exonic:treatment25
## treatment25:intronic
```

```
## treatment25:intergenic
## ---
## Signif. codes:  0 '***' 0.001 '**' 0.01 '*' 0.05 '.' 0.1 ' ' 1
##
## Correlation of Fixed Effects:
##               (Intr) exonic trtm25 intrnc intrgn exn:25 trtmnt25:ntrn
## exonic        -0.059
## treatment25   -0.277  0.071
## intronic      -0.021 -0.768  0.054
## intergenic    -0.045 -0.306  0.088 -0.267
## exnc:trtm25    0.025 -0.666 -0.092  0.523  0.205
## trtmnt25:ntrn  0.025  0.507 -0.091 -0.682  0.194 -0.761
## trtmnt25:ntrg  0.036  0.197 -0.122  0.191 -0.684 -0.306 -0.281
```

```
AIC(fit10a)
```

```
## [1] -2714.45
```
